# Injury-Induced Accumulation of Glial Cell Line-Derived Neurotrophic Factor in the Rostral Part of the Injured Rat Spinal Cord

**DOI:** 10.3390/ijms131013484

**Published:** 2012-10-19

**Authors:** Takuya Hara, Hidefumi Fukumitsu, Hitomi Soumiya, Yoshiko Furukawa, Shoei Furukawa

**Affiliations:** 1 Laboratory of Molecular Biology, Gifu Pharmaceutical University, Daigaku-nishi 1-25-4, Gifu 501-1196, Japan; E-Mails: tak-hara@kowa.co.jp (T.H.); hfukumi@gifu-pu.ac.jp (H.F.); somiya@gifu-pu.ac.jp (H.S.); 2 Department of Pharmaceutical Pharmacology, Faculty of Pharmaceutical Sciences, Matsuyama University, 4-2 Bunkyo-cho, Matsuyama, Ehime 790-8578, Japan; E-Mail: furukawa@cc.matsuyama-u.ac.jp

**Keywords:** glial cell line-derived neurotrophic factor (GDNF), spinal cord injury, enzyme immunoassay (EIA)

## Abstract

The spinal cord of a 7-week-old female Wistar rat was hemi-transected at thoracic position 10 with a razor blade, and changes in glial cell line-derived neurotrophic factor (GDNF) protein and mRNA expression levels in the spinal cord were examined. GDNF protein and mRNA expression levels were evaluated by enzyme immunoassay and reverse transcription polymerase chain reaction, respectively. Although GDNF is distributed in the healthy spinal cord from 150 to 400 pg/g tissue in a regionally dependent manner, hemi-transection (left side) of the spinal cord caused a rapid increase in GDNF content in the ipsilateral rostral but not in the caudal part of the spinal cord. On the other hand, injury-induced GDNF mRNA was distributed limitedly in both rostral and caudal stumps. These observations suggest the possibility that increased GDNF in the rostral part is responsible for the accumulation of GDNF that may be constitutively transported from the rostral to caudal side within the spinal cord. Although such local increase of endogenous GDNF protein may not be sufficient for nerve regeneration and locomotor improvement, it may play a physiological role in supporting spinal neurons including motoneurons.

## 1. Introduction

A limited regenerative response in the central nervous system (CNS) is a serious problem in mammals, although many experimental strategies have been employed to minimize tissue damage and enhance axonal growth and regeneration. In the case of spinal cord injury (SCI), the failure of axonal regeneration is thought to result partly from the lack of neurotrophic factors [1–3], in addition to expression of axonal growth-inhibiting molecules [4] and/or inflammatory reactions [5]. In particular, neurotrophic factors such as neurotrophins and glial cell line-derived neurotrophic factor (GDNF) have been reported to be beneficial for axonal regeneration when applied to the injury site of the spinal cord [1,6–8]. Furthermore, recent reports have shown that endogenous GDNF in the spinal cord plays a role in nerve regeneration and contributes to improved locomotor activity to some extent after spinal cord injury because nerve regeneration and locomotor activity are weakened by intraperitoneal administration of an anti-GDNF antibody [9]. We found that high-dose lipopolysaccharide improved locomotor function to a greater extent than low-dose lipopolysaccharide, consistent with expression of GDNF in microglia/macrophages of the injured spinal cord [2,3]. In addition, experiments using GDNF gene mutant mice confirmed that increase in GDNF expression levels and no reduction in mRNA levels of inducible nitric oxide (NO) synthase correlate with restoration of locomotor function [3]. These results suggest that a high degree of inflammation results in higher amelioration of spinal cord injury through facilitated production of GDNF and endogenous GDNF plays a critical role in amelioration of SCI. However, such molecular events only occur in a limited manner in the CNS [10], resulting in a poor regeneration and locomotor improvement that characterizes SCI. Investigations regarding the role of endogenous GDNF in the spinal cord following injury are important in determining the effects of neurotrophic factors on amelioration of the injury.

Therefore, in the present study we examined changes in GDNF protein and mRNA expression levels in the spinal cord after hemi-transection and found that without affecting the expression level of mRNA, its translation product was increased in the rostral side from lesion site, suggesting the plausible possibility that constitutively transportation of GDNF protein from the rostral to caudal side in the spinal cord. Although, local increase of endogenous GDNF protein may not be sufficient for nerve regeneration and locomotor improvement, it may play a physiological role in supporting spinal neurons including motoneurons.

## 2. Results

### 2.1. Distribution of GDNF in Intact Spinal Cord

**Figure 1:**
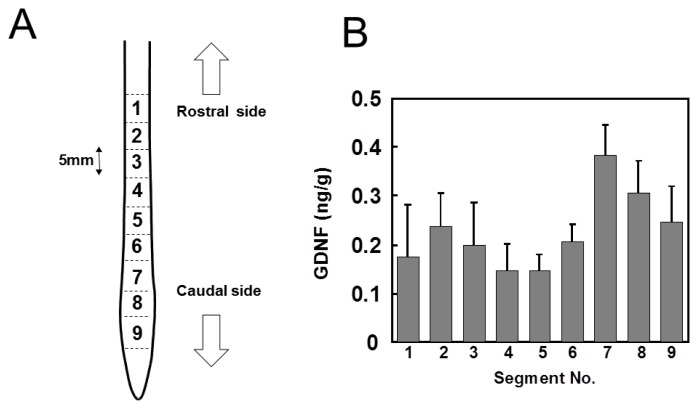
Glial cell line-derived neurotrophic factor (GDNF) protein distributes differentially in concentration by position in the spinal cord of adult rats. (**A**) The spinal cords were dissected from normal adult rats and cut into nine segments of 5 mm length. Arrows indicate the rostral and caudal sides. (**B**) Each segment was pulse-sonicated, centrifuged, and the supernatant fluids were subjected to enzyme immunoassay (EIA). The values are represented as mean ± SE of five animals.

The detection limit of our enzyme immunoassay (EIA) system was as low as 5 pg/mL and the detection signal was dose-dependent below 1000 pg/mL, demonstrating that EIA was satisfactory for the measurement of endogenous GDNF in the injured or intact spinal cord. Furthermore, it has been confirmed that our EIA system is specific for GDNF among GDNF family proteins including artemin, neurturin, and percephin [11]. The recovery of exogenously added GDNF from the tissue homogenate was 76.7%, which confirmed the effectiveness of the measurement. Therefore, the values thus obtained by the EIA were used without correction. The intact spinal cord was dissected and cut into 9 segments of 5 mm length. The segments were numbered rostrocaudally from 1 to 9 (Figure 1A). GDNF content in each segment varied from 150 to 400 pg/g wet tissue weight (Figure 1B). The segments of the lumbar region (Nos. 7, 8, and 9) may contain much higher concentrations of GDNF than segments of other regions.

### 2.2. Changes in GDNF Protein Content after SCI

**Figure 2:**
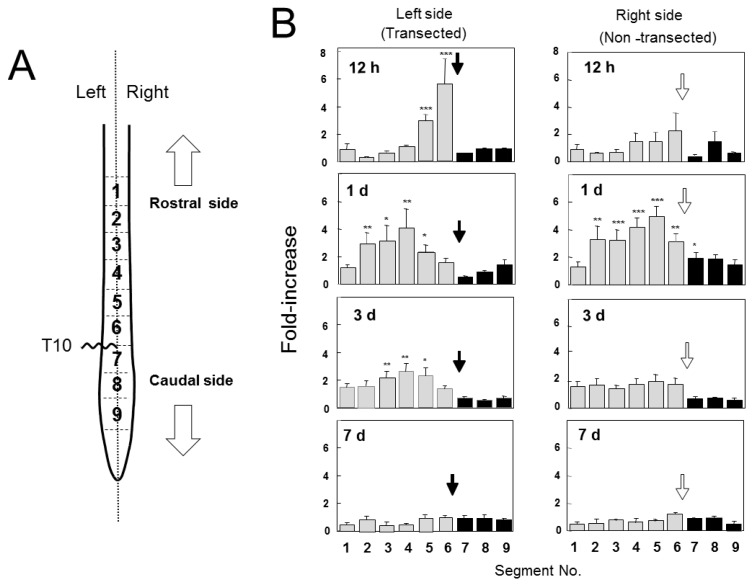
Spinal cord injury (SCI) increases GDNF protein in the rostral part of the spinal cord. (**A**) The wavy line indicates the hemi-transection site at T10. Arrows indicate the rostral or caudal side. The spinal cords were dissected at the indicated time and divided in the middle into the injured (**left**) or non-injured (**right**) side. Both were cut into nine segments of 5 mm length. (**B**) Each segment was pulse-sonicated, centrifuged, and the supernatant fluids were subjected to EIA. Values are represented as mean ± SE of 3–8 animals. Closed arrows on the left side indicate the transected position, and open arrows on the right side indicate the position corresponding to the contralateral transection site. Significance: * *p* < 0.05, ** *p* < 0.01, *** *p* < 0.001 *vs*. the value of corresponding segment of normal animals (Student’s *t*-test).

We examined GDNF content in the spinal cord after hemi-transection at T10 (Figure 2). In the transected side (left side), a marked increase in GDNF protein occurred in the rostral stumps that were adjacent or near to the injury site (segments Nos. 5 and 6) 12 h post injury (POI). The increase extended to more segments (Nos. 2–5) 1 day POI, and slightly reduced to 3 segments (Nos. 3–5) 3 days POI. The GDNF content gradually decreased 3 days POI and recovered to original levels by 7 days POI. However, no increase was observed in the caudal stumps throughout the experiments. In contrast, in the uninjured side (right), GDNF levels were not significantly high in all segments 12 h POI; however, its levels peaked in many rostral segments (segment Nos. 2–6) 1 day POI. GDNF levels returned to normal levels by 3 days POI. These results clearly demonstrated that injury-induced increase in GDNF content was conspicuous in the rostral part, but not in the caudal part, in both the injured and uninjured sides of the spinal cord.

It is likely that the non-transected side responded to the contralateral transection similarly but weakly and after a longer period of time [12,13]. It may be possible that the transection of half of the spinal cord influenced the other half by rapid gathering of macrophages/microglia to cause inflammation. The inflammation might stop axonal flow carrying GDNF protein from the rostral to caudal side, which action could result in the accumulation of GDNF at segment 6 and withdrawal of it at segment 7, thus accounting for the increase and decrease in segment 6 and 7, respectively. Since the response caused by the inflammation may be considered to be somewhat weaker and slower than the response to the transection, the increase in segment 6 of the uninjured side would be smaller than that in the corresponding segment of the injured side.

There are several possible reasons for the injury-induced increase of GDNF only in the rostral part of the spinal cord: (1) *de novo* synthesis of GDNF, (2) accumulation of GDNF being transported from rostral to caudal side of the spinal cord, (3) accumulation of GDNF being retrogradely transported within the peripheral nerves from the skeketal muscles to the motoneurons, (4) binding of GDNF to the injury-evoked GFRα1, coreceptor of GDNF, (5). supply of GDNF from the dorsal root ganglia (DRG).

**Figure 3:**
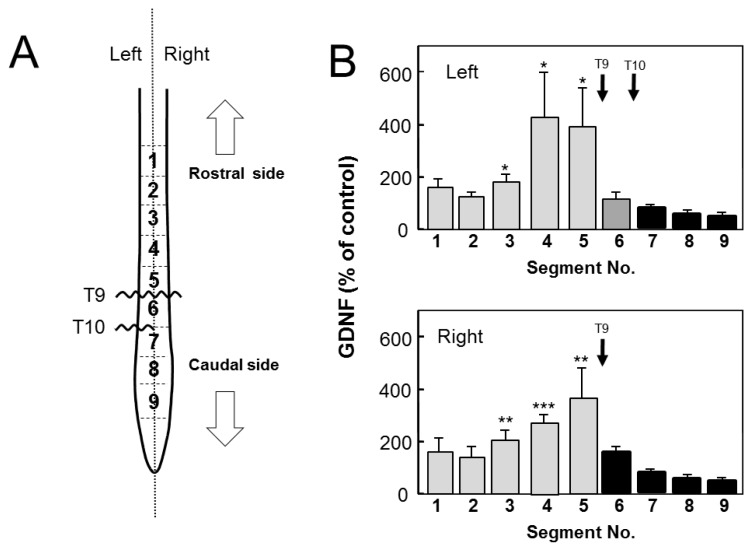
Two-point-transection model confirms a lack of GDNF transport from the caudal to rostral side. (**A**) Wavy lines indicate a complete transection site at T9 and a hemi-transection site at T10. Arrows indicate the rostral or caudal side. The spinal cords were similarly processed as described in the legend of Figure 2A. (**B**) Each segment was treated similarly as described in the legend of Figure 2B. Values are represented as mean ± SE of five animals. Arrows indicate the position of transection. Significance: * *p* < 0.05, ** *p* < 0.01, *** *p* < 0.001 *vs*. the value of corresponding segment of normal animals (Student’s *t*-test).

The forelimbs may compensate for acute functional loss of the hindlimbs which may require more *de novo* synthesis of GDNF in the cervical enlargement. Therefore, GDNF protein might be synthesized *de novo* in a physical activity-dependent manner. To clarify possibilities (1), (2) and (5), we prepared an animal model with injuries of both a complete transection at T9 and a left side hemi-transection at T10 (Figure 3A). We separately measured GDNF content in the left and right side of each segment 12 h POI. If there was injury-induced enhancement of *de novo* synthesis in the rostral side, increase in GDNF content could occur in segment 5 and 6 of the left side and segment 5 of the right side, because they were rostral segments adjacent to the injury sites. The left and right segments 5 were similarly increased, but not the left segment 6 (Figure 3B), which could be explainable by a possibility that GDNF transporting from rostral to caudal side within axons was accumulated at segments 5 by interference with its transport. Furthermore, this result clearly confirmed that GDNF does not accumulate in the caudal stumps, suggesting that GDNF transport from caudal to rostral part does not function in the spinal cord (Figure 3B).

The possibility (5), supply of GDNF from the DRG, should be considered as a reason for the injury-induced rostral accumulation of GDNF. The DRG neurons project axons to relay neurons of specific laminae in the dorsal area of the gray matter of the spinal cord. A recent report [14] demonstrated that when afferent axons from the DRG reach the dorsal root entry zone of the spinal cord during embryonic development, they display a stereotyped pattern of T- or Y-shaped bifurcation. Therefore, DRG axons penetrate straightly into the dorsal root entry zone and form this bifurcation in the spinal cord to connect to target neurons. The anatomy of axons of DRG neurons is important to estimate the possibility of the present issue at hand. Hemitransection of the spinal cord might injure DRG neurons and consequently interfere with anterograde axonal transport of GDNF, resulting in accumulation of GDNF within these axons. However, it should be noted that axons of DRG neurons penetrate perpendicularly to the spinal cord, and orthogonally to the axons that run up and down within the cord. Therefore, only DRG axons in the vicinity of the hemitransection site would be injured and accumulate GDNF being transported within them. DRG axons apart from the injury site would function normally and show no change in GDNF distribution. If the axons of DRG neurons were injured by transection of the spinal cord, GDNF from the DRG would have accumulated around the injury sites in both rostral and caudal sides of all injury sites to which DRG axons project. Therefore, if this were the case, the two-point transection model shown in Figure 3 would have given different results. Assuming that injury to the axons of DRG neurons was responsible for GDNF accumulation in the spinal cord, then all segments both rostral and caudal to the transection sites should have contained a substantial level of GDNF. From these results and considerations, possibility (5) was excluded.

However, another explanation that the neurons located in left segment 6 which synthesized GDNF *de novo* would die following double transaction injury due to progressive disruption of long axon tracts and extensive tissue loss might be possible. As this possibility was based on the injury-enhanced neuronal GDNF synthesis, we evaluated GDNF mRNA expression in the all segments including left segment 6 after hemi-transection.

### 2.3. GDNF mRNA Expression after Spinal Cord Injury

**Figure 4:**
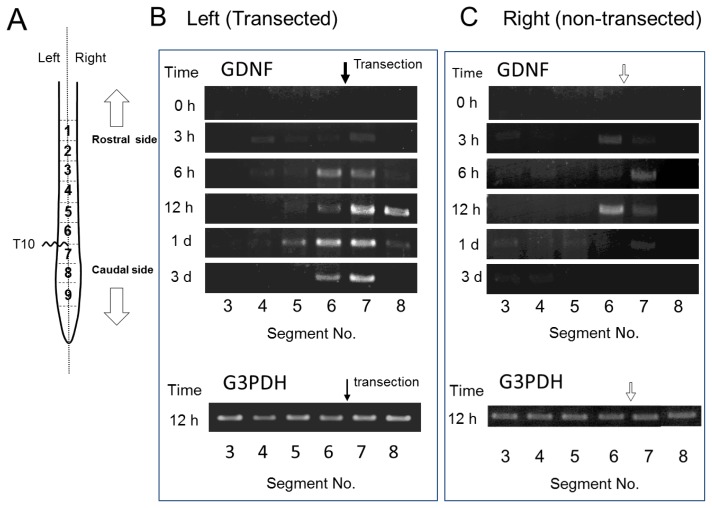
SCI induces GDNF mRNA expression in the rostral and caudal stumps adjacent to the injury sites. (**A**) Same as described in the legend of Figure 2A. (**B** and **C**) Total RNA was isolated from each segment using TRIZOL Reagent (Invitrogen) according to the manufacturer’s instructions. All RNA samples were treated with DNase I to remove contaminating genomic DNA. RT-PCR was performed to assess the GDNF mRNA level using G3PDH mRNA as the internal control. A closed arrow in (**B**) indicates transection site of the spinal cord, and an open arrow in (**C**) demonstrates the corresponding position to the contralateral transection site.

We evaluated GDNF mRNA expression in each segment after SCI by RT-PCR (Figure 4). In the transected left side, GDNF mRNA was evenly detected in both rostral and caudal stumps adjacent to the injury site 6 h POI onset until at least 3 days POI examination. In the non-transected right side, the expression was similarly detected in both rostral and caudal stumps; however, the expression was weaker and more transient compared with that in the left side probably because of the low severity of the injury. These results demonstrated a mismatch of GDNF mRNA and GDNF protein in their distribution after SCI. Therefore, enhanced *de novo* synthesis of GDNF was unlikely as a reason for rostral GDNF increment, because of the inconsistency between mRNA expression and GDNF protein levels. Therefore, it is possible that marked increase in GDNF protein in the rostral side may be responsible for the accumulation of GDNF protein that is transported within the spinal cord. We did not provide the results on mRNA expression in the sham-operated controls because it was almost the same as the results for the “control (0 h after SCI)” shown in Figure 4. Namely, GDNF mRNA expression was weak or lacking in the sham-operated uninjured spinal cord, suggesting that the substantial level of GDNF protein detected in the spinal cord (Figures 1 and 2) was not largely responsible for *de novo* synthesized GDNF in the spinal cord. This possibility supports constitutive transport of GDNF within the spinal cord.

### 2.4. GFRα1 mRNA Expression after Spinal Cord Injury

The injury-induced expression of GDNF receptors [15] might cause GDNF increase in the rostral part, enhancing binding and accumulation of GDNF in this region. We examined time course of regional mRNA expression of GFRα1, GDNF binding coreceptor, after hemi-transection. GFRα1 mRNA increased rapidly from as early as 3 h POI in almost all segments of both transected and non-transected sides, and the increment continued till 3 days POI. Therefore, we noticed that the injury-induced enhancement of GFRα1 mRNA expression occurred in extensive, not limited to the rostral part of the injury site, suggesting that GFRα1 was not involved in mechanisms for rostral GDNF accumulation.

**Figure 5:**
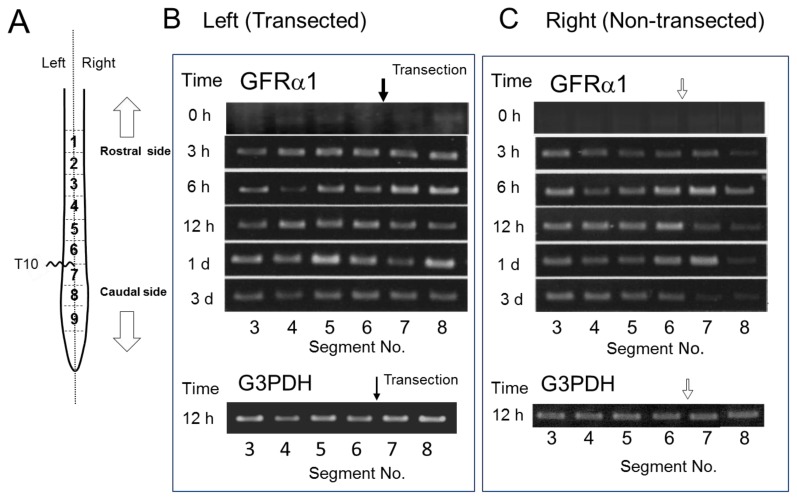
SCI-induced GFRα1 mRNA expression in the rostral and caudal stumps. (**A**) As described in the legend of Figure 2A. (**B** and **C**) RT-PCR was performed to assess the GFRα1 mRNA level as described in the Figure 4 legend using G3PDH as the internal control.

We expected at first that hemi-transection of the spinal cord would make a great difference between ipsilateral and contralateral sides. Contrary to this expectation, both sides showed a similar change, although response of the non-transected (right) side was somewhat weak and slow. However, the rapid increase of GDNF in rostral side was observed only in the transected left side. In over one day, both sides showed a similar GDNF distribution. It is likely that the transection of half of the spinal cord influences on the contralateral half by rapid gathering of macrophages/microglia to cause inflammation. Our present results demonstrated that injury-induced GFRα1 mRNA expression occurred similarly in both transected and non-transected sides as early as 3 h after the injury, suggesting that hemi-transection caused similar influence on the transected and non-transected side (Figure 5).

### 2.5. GDNF Immunoreactivity in Intact and Injured Spinal Cord

**Figure 6:**
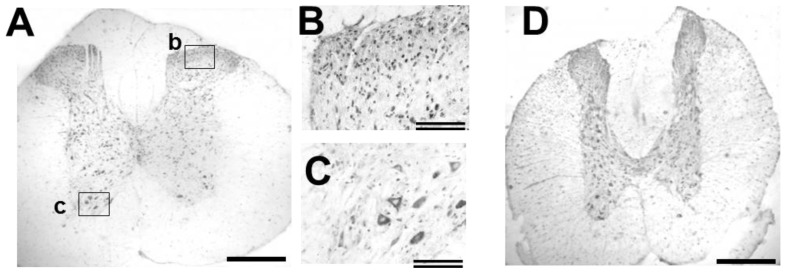
GDNF-ir in intact and injured spinal cord. GDNF-ir in coronal sections of the intact (**A**, **B**, **C**, site around T9-10) or injured (**D**, rostral part to the injury site at T9-10, 12 h post injury (POI)) spinal cord was shown in low- (**A**, **D**) and high-power photographs (**B**, **C**). (**B**) and (**C**) are enlargements of boxes “b” and “c” in (**A**), respectively. Scale bars: single line, 500 μm; double line, 100 μm.

Distribution of GDNF-immunoreactivity (ir) in the intact spinal cord is shown in low- and high-power photographs (Figure 6). GDNF-ir was observed in the cell bodies of motoneurons and in small cell bodies of the dorsal area. The antibody preabsorbed with peptide containing the antigen sequence gave no significant signal (data not shown), demonstrating antibody specificity for GDNF. Our present results (Figure 4C) demonstrated that GDNF mRNA expression was low in the intact spinal cord when compared to the expression around the injury site, supporting the fact that GDNF mRNA was undetectable in motoneurons [16]. In contrast, GDNF generates signals through a receptor complex comprising coreceptors (GFRα1) and Ret tyrosine kinase. GFRα1 mRNA is known to be expressed in spinal neurons, including motoneurons [17], and widely distributed in the whole spinal cord. Furthermore, another molecule for signal generation, c-Ret, is also expressed in motoneurons [18,19]. Therefore, it is likely that GDNF-ir found in spinal cells, including motoneurons, was derived at least partly from GDNF synthesized elsewhere and bound to the neurons.

To obtain evidence that GDNF is transported and accumulated in the rostral side, we examined the distribution of GDNF-ir around the rostral stumps. As shown in Figure 5D, GDNF-ir did not show any obvious change in distribution and/or intensity. Therefore, we could not obtain such evidence, but exclude a possibility that GDNF being retrogradely transported within the peripheral nerves from the skeketal muscles to the motoneurons.

A question why the GDNF-ir was less clear in the immunohistochemical analysis than the EIA may arise. This may be due to differences in the principle of the detection. That is, the GDNF transporting within the axons was thought to be biologically active and diffusible form. Such moleculesm might be easily extracted from the unfixed tissues, and might be released unexpectedly from the fixed tissues. Therefore, detection efficiency of GDNF by immunohistochemical technique might be lower than it was. In this point of view, EIA is reliable to quantify active and diffusible GDNF molecules.

### 2.6. Injury-Induced GDNF Synthesis in Immune Cells

**Figure 7:**
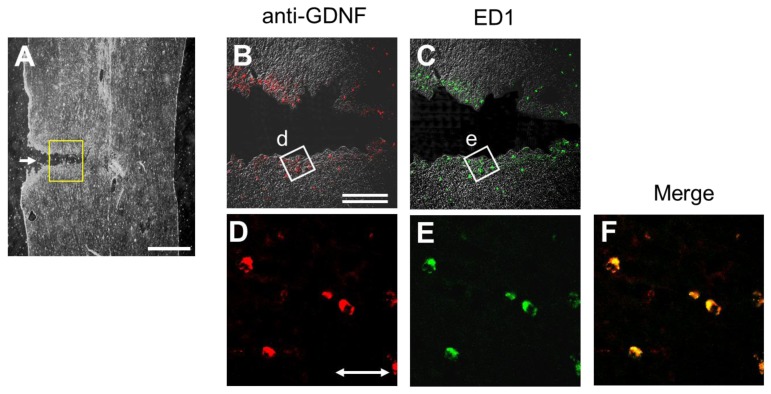
GDNF-ir-positive cells near the injury site are bearing ED-1 antigen. GDNF-ir (red color) (**A**, **B**, **D**) and ED-1 antigen (green color) (**C**, **E**) in sagittal sections of the injured spinal cord were shown in low- (**A**), middle- (**B**, **C**) and high-power photographs (**D**, **E**). It was shown that GDNF-ir-positive cells present in the rim of the injury site expressing ED-1 antigen, a specific marker for microglia/macrophages. The box in (**A**) was enlarged into (**B**) and (**C**). The box “d” in (**B**) and the box “e” in (**C**) were enlarged into (**D**) and (**E**), respectively. Scale bars: single line, 500 μm; double line, 100 μm; line with arrowheads, 20 μm.

Another type of GDNF-ir-positive cell was detected in both the rostral and caudal rims of the transection site. Most of these cells co-expressed ED-1 antigen and GDNF-ir (Figure 7), demonstrating that they were microglia or macrophages. This GDNF-ir expression by microglia/macrophages was timely and regionally coincident with the injury-induced mRNA expression observed in both the rostral and caudal stumps adjacent to the injury site (Figure 4C). However, amount of GDNF synthesized by the immune cells was thought to be small, because the EIA for GDNF could not detect elevations in GDNF levels of the caudal stumps, except for segment 7 one day POI with significant increase (Figure 2).

## 3. Discussion

GDNF, which generates signals through a receptor complex comprised of the coreceptors, (GFRα1) and c-Ret tyrosine kinase, which activate phosphoinositide 3-kinase and mitogen-activated protein kinase pathways to enhance cell survival, lamelopodia formation, and axonal elongation, provides trophic cues to midbrain dopaminergic neurons and spinal motor neurons in central nervous system (CNS) [16,17,20,21]. GDNF signaling has also been shown to regulate other aspects of motoneuron development, including the expression of motoneuron subtype-specific transcription factors, motor axon projections, and muscle innervations [16,18,22–25]. Widespread expressions of GDNF in developing skeletal muscles are consistent with neurotrophic activity in motoneurons of the spinal cord [26]. Furthermore, a recent report revealed that GDNF induces BDNF gene expression, which suppresses apoptosis of nigrostriatal dopaminergic neurons through the transcription factor Pitx3 [27].

GDNF mRNA is mainly distributed in neurons in various CNS regions, including the cortex and spinal cord [28,29]. GDNF-ir is observed widely in the CNS of humans [30] and rodents [31]. For one example, Tokumine *et al*. [32] detected GDNF protein in the spinal cord by EIA as 74 ± 22 pg/g tissue weight, which is slightly lower but comparable to the values in our study, as shown in Figure 1. Ischemia increased GDNF levels in the spinal cord reaching two peaks [33] derived from a-motoneurons and astrocytes 2 h and 72 h after onset of recirculation, respectively, suggesting that activated astrocytes may have a role in maintaining high levels of GDNF. However, the expression of GDNF mRNA and protein is known to be regulated in a cell-, region- or insult-specific manner [34]. Therefore, the physiological roles of GDNF should be examined individual situation.

In the present study, we examined SCI-induced changes in expression and distribution of GDNF protein and mRNA within the spinal cord. GDNF content rapidly increased and was regionally distributed in the rostral part but not the caudal part (Figure 2); however, GDNF mRNA was expressed only at both the rostral and caudal sites (Figure 4C). ED-1-positive cells, microglia/macrophages, were distributed around the rim of the injury site and expressed GDNF-ir, as shown in Figure 7. It should be noted that ED-1-positive cells were similarly distributed as putative GDNF mRNA-expressing cells, suggesting that microglia/macrophages were the major cells producing GDNF in the proximal region to the lesion site. Indeed, our previous quantitative analysis demonstrated that almost all of the GDNF-positive cells expressed ED-1 12 h or 2 days POI [2,3]. The hypothesis is supported by the fact that GDNF protein was not increased in the segment 6 in the spinal cord of T9/T10-level double transections (Figure 3). In other word, GDNF may be always transporting from the rostral to caudal side within the nerve tracts and accumulates in the rostral side by SCI-induced interference of transport. Although we did not know the physiological roles of the accumulated GDNF, such GDNF may contribute to neurotrophic cues such as support of survival and maintenance of function of some spinal neurons including motoneurons. By using GDNF heterozygous mice, we have previously demonstrated that deficiency of endogenous GDNF significantly compromise the function recovery from hemi-transection of spinal cord [3].

Recent reports have shown that recombinant adeno-associated virus (rAAV)-derived GDNF can be delivered to the lumbar spinal cord through the pathways of upper motoneurons, the corticospinal tracts (CST), and rubrospinal tracts (RST) over considerable distances by anterograde transport [35]. Furthermore, it has been demonstrated that rAAV5-GDNF gene infections in the red nucleus resulted in GDNF-positive fibers projecting into spinal gray matter; however, cortical infections drew less evident staining in the spinal cord. These observations imply that GDNF is anterogradely transported from the rostral to caudal side, predominantly originating from the RST tracts rather than the CST tract, which may support our present results showing a rapid and regional accumulation of GDNF in the rostral side after SCI. Alternatively, retrograde axonal transport of GDNF from the myofibers to motoneurons in the rostral stumps may be enhanced after SCI. In fact, rAAV-derived GDNF was substantially expressed and distributed in a large number of myofibers, mainly in the vicinity of the sarcolemma after intramuscular infection of the rAVV-GDNF gene and predominantly concentrated at the sites of neuromuscular junctions [36], suggesting retrograde axonal transport of GDNF from muscle to motoneurons. In any case, GDNF accumulated in the rostral stump after SCI may participate as neurotrophic factors to facilitate nerve regeneration of CST and/or RST.

Injury-induced alterations in localization and distribution of neurotrophic factors were observed in the sciatic nerve [37–40]. In particular, nerve growth factor, a prototype of neurotrophins, is known to be retrogradely transported within the sciatic nerve from peripheral end organs/tissues to the dorsal root and/or sympathetic neurons as a target-derived neurotrophic factor [37,40]. The transection of the sciatic nerve caused a rapid and marked increase in NGF protein levels, only in the distal stump adjacent to the injury site within 1 day POI, and this increase occurred independent of mRNA expression. This was probably due to the fact that the machinery for axonal transport worked for a while after nerve transection and resulted in the accumulation of transporting NGF. NGF synthesis gradually started broadly in the distal parts of the injured sciatic nerve. NGF accumulated in the distal stumps was used for sprouting and extension of axons from the proximal stump. Similarly, the axonal flow of GDNF would gradually weaken 12 h and not likely to last more than 3 days after transection (Figure 2B). The transient axonal flow not followed by local production of GDNF protein may result in a poor nerve regeneration or insufficient locomotor improvement that characterizes SCI. In the peripheral nervous system, we previously examined the changes in GDNF content in the sciatic nerve after its transection, and found that GDNF protein transporting in the sciatic nerve may be too small to accumulate in the injury site; however, the injury caused a high level of *de novo* GDNF synthesis [11]. Injury-activated Schwann cells in the distal areas expressed GDNF mRNA to produce large amounts of GDNF protein, which were negatively regulated by interaction with axonal contact [11].

Information about distribution of GDNF before and after injury may be important in understanding the physiological roles of GDNF. Zhou *et al*. [9] showed that GDNF in the spinal cord may be involved in the mechanisms underlying nerve regeneration and recovery of locomotor activity because these ameliorative effects are lost by an anti-GDNF antibody. They demonstrated that GDNF protein was more abundant in rostral part than caudal part at 3 days after operation, but in similar level in both parts at 7 days by Western immunoblot. These results were well coincident with ours obtained by the EIA at 3 and 7 days after operation (Figure 2). However, Zhou *et al*. did not analyze at 12 h and 1 day after operation, which failed to compare with our results at those times. Eventually, their results obtained by Western immunoblot are well correlated with our results obtained by the EIA, supporting our idea that GDNF synthesized in the red nucleus and/or somatosensory cerebral cortex is anterogradely transported from the rostral side to caudal side within the nerve tracts in the spinal cord as a neurotrophic factor. Such GDNF may act on spinal neurons, including motoneurons and/or neurons of the red nucleus and cerebral cortex and GDNF-synthesizing neurons in an autocrine fashion. Therefore, injury-induced accumulation in the rostral side of GDNF may contribute to reduced spasticity and improved functional outcomes in SCI [41].

## 4. Experimental Section

### 4.1. Animals and Surgery

Animals were handled in accordance with the Guidelines of Experimental Animal Care issued by the Office of the Prime Minister of Japan. All efforts were made to minimize the number of animals used. Female Wistar rats (7 weeks old, *n* = 51 animals, Nippon SLC) were anesthetized with pentobarbital (35 mg/kg i.p.), and the spinous process and the vertebral lamina were removed to expose the whole cord at the laminectomy site. Half of the spinal cord (left side) was then transected at thoracic position (T) 10 with a razor blade. After surgery, the superficial back muscles were sutured along the midline and the skin was closed with Michel wound clips. The animals were fed for various periods of time and then anesthetized again. The left side or right side of the spinal cord was separately dissected from each animal, cut into 6 pieces of 5 mm in length and stored at −80 °C until subsequent analysis.

### 4.2. Preparation of Tissue Samples

Each sample was pulse-sonicated in 5% (*w*/*v*) 0.1 M Tris-HCl buffer, pH 7.6, containing 1 M NaCl, 0.1% bovine serum albumin (BSA), 2 mM EDTA, 80 trypsin inhibitor units/L aprotinin (Sigma-Aldrich, St. Louis, MO, USA), and 0.02% NaN_3_ and centrifuged at 100,000× *g* for 30 min. The supernatant was mixed with an equal volume of chloroform to remove lipids and centrifuged again at 20,000× *g* for 30 min, following which the aqueous phase was used for the measurement of GDNF protein.

### 4.3. Measurement of GDNF Content by Enzyme Immunoassay

Enzyme immunoassay (EIA) was performed according to a method described previously [11]. In brief, anti-GDNF antiserum was prepared from rabbits immunized with recombinant GDNF (provided by Amgen Inc., Thousand Oaks, CA, USA), and anti-GDNF antibody was purified from the antiserum using a GDNF-linked column (Affi-gel 10; Bio-Rad, Hercules, CA, USA). Affinity-purified anti-GDNF antibody (10 mg/mL) in 0.1 M Tris-HCl buffer (pH 9.0) was coated onto the well of 96 U-bottom multiwell plates (5 mL/well) and incubated at 25 °C for 2 h. For evaluation of the background signal, control wells were treated with normal rabbit IgG. The wells were washed with 0.1 M Tris-HCl buffer (pH 7.6) containing 0.4 M NaCl, 0.1% BSA, 1 mM MgCl_2_, and 0.02% NaN_3_ (washing buffer), and non-occupied space was blocked by incubation with 100 mL/well of 1% (*w*/*v*) skim milk for 1 h. After washing, 30 mL of test sample or serially diluted recombinant GDNF was incubated in the wells for 2 h at 25 °C. Then, 30 mL of affinity-purified biotinylated antibody (10 ng/mL) diluted in the washing buffer was incubated for 12–18 h at 4 °C. Finally, 30 mL of β-d-galactosidase-conjugated streptavidin was added to each well. After incubation at 25 °C for 1 h, bound β-d-galactosidase activity was measured by incubation with 30 mL of 30 mM 4-methylumbelliferyl-β-d-galactoside. The intensity of fluorescence was monitored at 360 nm excitation and 448 nm emission (Model F-2000, Hitachi, Tokyo, Japan). The standard curve of GDNF was used for determination of concentrations.

### 4.4. Immunohistochemical Procedures

Rats were anesthetized and successively perfused with cold phosphate-buffered saline (PBS, 50 mL) and cold 4% (*w*/*v*) paraformaldehyde solution prepared in 0.1 M phosphate buffer, pH 7.3 (200 mL). In the case of injured rats, 5 mm sections of the spinal cord were cut out from the transection site rostrally or caudally. These segments were postfixed for 2 h in the same fixative and frozen in embedding compounds (Miles, Elkhart, IN, USA). Coronal sections of 20-μm thickness were prepared with a cryostat (Model CM 1800, Leica, Wetzlar, Germany). For immunostaining, sections were rinsed in 0.1 M Tris-HCl buffer, pH 7.6, containing 0.3% (*v*/*v*) Triton X-100 (TT buffer) at 4 °C for 1 day to render the cell membrane permeable to antibodies and incubated with anti-GDNF rabbit antibody (0.3 μg/mL; d-20, Santa Cruz Biotechnology Inc., Santa Cruz, CA, USA) or anti-CD68 antibody (ED1) to recognize the antigen of microglia/macrophages (0.5 μg/mL; Antigenix America Inc., Huntington Station, NY, USA) in TT buffer at 4 °C for 1 day. The epitope of the antibody (D20) was mapped near the *C*-terminus of GDNF of human origin and recommended for detection of mouse, rat, and human GDNF. The sections were successively treated with 0.3% (*v*/*v*) H_2_O_2_ at 25 °C for 30 min to quench endogenous peroxidase activity, and 2% skim milk to minimize non-specific binding. They were further reacted with rhodamine-conjugated goat anti-rabbit IgG (Invitrogen, Carlsbad, CA, USA) and fluorescein isothiocyanate (FITC)-conjugated goat anti-mouse IgG (Invitrogen). Images were observed with a confocal laser microscope (LSM 510, Zeiss, Oberkochen, Germany).

### 4.5. Reverse Transcription-Polymerase Chain Reaction (RT-PCR)

RT-PCR was performed as described [3]. The specific primers used were as follows: forward primer 5′-GAGAGGAATCGGCAGGCTGCAGCTG-3′ and reverse primer 5′-CAGATACATCCACATCGTTTAGCGG-3′ for GDNF (product size: 337 bases); forward primer 5′-CCGGGCAGTCCCGTTCATA-3′ and reverse primer 5′-TCAGTCCCGAGTAGGCCAGGAG-3′ for GNRα1 (product size: 482 bases); and forward primer 5′-CGGAGTCAACGGATTTGGTCGTAT-3′ and reverse primer 5′-AGCCTTCTCCATGGTGGTGAAGAC-3′ for glyceraldehyde-3-phosphate dehydrogenase (G3PDH) (product size: 309 bases). The G3PDH gene was used as an internal control. After amplification, PCR products were subjected to 2% agarose gel electrophoresis and visualized by ethidium bromide staining. Images were captured with FLA-5100 (Fuji Film, Tokyo, Japan).

### 4.6. Statistical Analyses

Data are presented as mean ± SE. The statistical significance of the differences between the two groups was assessed using Student’s *t-*test.

## 5. Conclusions

GDNF is distributed in the spinal cord from 150 to 400 pg/g tissue in a regionally dependent manner. Hemi-transection of the spinal cord (right side) caused a rapid increase in GDNF content in the rostral but not the caudal part of the spinal cord; however, injury-induced GDNF mRNA was distributed evenly in both rostral and caudal stumps. Collectively, our present results suggest that increased GDNF in the rostral part is responsible for the accumulation of GDNF that is constitutively transported from the rostral to caudal side within the spinal cord, and may contribute to nerve regeneration and improvement of locomotor activity.
